# Boosting Light‐Driven CO_2_ Conversion Into CO by a Polypyridine Iron(II) Catalyst Using an Organic Sensitizer

**DOI:** 10.1002/cssc.202402627

**Published:** 2025-02-06

**Authors:** Federico Droghetti, Lucrezia Villa, Andrea Sartorel, Luca Dell'Amico, Albert Ruggi, Mirco Natali

**Affiliations:** ^1^ Department of Chemical, Pharmaceutical and Agricultural Sciences (DOCPAS) University of Ferrara Via L. Borsari 46 44121 Ferrara Italy; ^2^ Department of Chemical Sciences University of Padova Via F. Marzolo 1 35131 Padova Italy; ^3^ Department of Chemistry University of Fribourg Chemin du Musée 9 1700 Fribourg Switzerland

**Keywords:** CO_2_ reduction, Iron complex, Thermally-activated delayed fluorescence, Homogeneous catalysis, Selectivity

## Abstract

Direct photochemical conversion of CO_2_ into a single carbon‐based product currently represents one of the major issues in the catalysis of the CO_2_ reduction reaction (CO_2_RR). In this work, we demonstrate that the combination of an organic photosensitizer with a heptacoordinated iron(II) complex allows to attain a noble‐metal‐free photochemical system capable of efficient and selective conversion of CO_2_ into CO upon light irradiation in the presence of *N*,*N*‐diisopropylethylamine (DIPEA) and 2,2,2‐trifluoroethanol (TFE) as the electron and proton donor, respectively, with unprecedented performances (Φ_CO_ up to 36 %, TON_CO_ >1000, selectivity >99 %). As shown by transient absorption spectroscopy studies, this can be achieved thanks to the fast rates associated with the electron transfer from the photogenerated reduced dye to the catalyst, which protect the dye from parallel degradation pathways ensuring its stability along the photochemical reaction. These results point out how the profitable merging of molecular species based on cheap and abundant elements can have great potential to target efficient and selective transformations crucial for the conversion of solar energy into fuels.

## Graphical abstract

The combination of a thermally‐activated delayed fluorescence (TADF) dye with a heptacoordinated iron complex as a catalyst allows to achieve a noble‐metal‐free photochemical system capable of boosting light‐driven catalytic conversion of CO_2_ into CO with unprecedented (quantum) efficiencies and almost unitary selectivity.

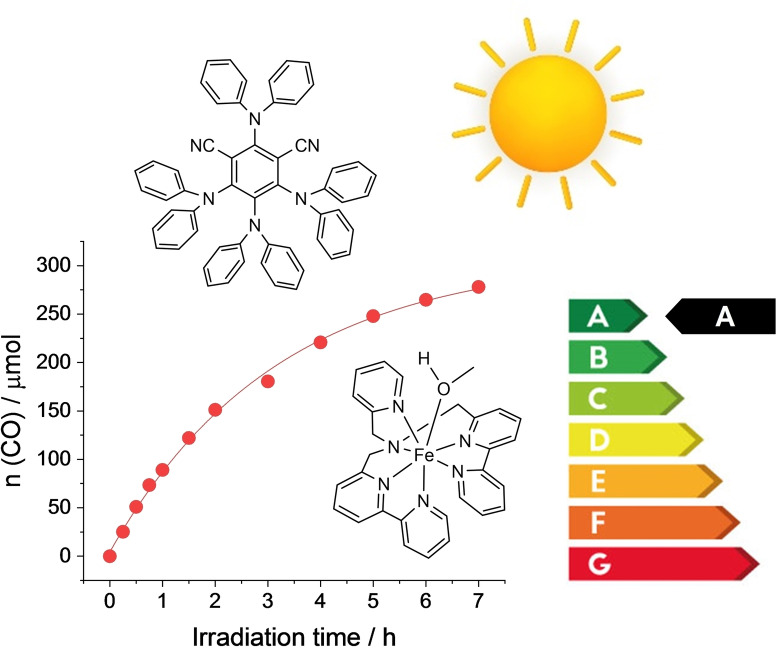

## Introduction

Light‐driven catalysis of the CO_2_ reduction reaction (CO_2_RR) currently appears as one of the most interesting opportunities within the framework of Artificial Photosynthesis (AP).[^1^, ^2^, ^3^] Direct conversion of CO_2_ into carbon‐based products is, however, not a trivial task, particularly due to the involvement of multiple proton‐ and electron‐transfer steps.[^4^, ^5^] These lead to significant selectivity issues ensuing from the kinetic competition among different processes and the parallel hydrogen evolving reaction (HER). Inspired from the active sites of natural enzymes, many metal complexes have been designed and employed as catalysts to target efficient and selective CO_2_RR to carbon‐based products, mainly resulting in the preferential generation of CO and/or formate.[^6^, ^7^, ^8^, ^9^, ^10^] Within this framework, polypyridine complexes of first‐row transition metals have received particular attention, as they combine high activity and remarkable stability under turnover conditions.^[10]^ Most of these molecular catalysts have been also effectively combined with specific light‐harvesting components and electron donors to attain direct conversion of CO_2_ into products using light.[^11^, ^12^, ^13^, ^14^, ^15^] In this regard, remarkable performances were attained in acetonitrile combining, e. g., a [Co(qpy)(OH_2_)_2_]^2+^ (where qpy=quaterpyridine) catalyst with the triazatriangulenium sensitizer^[16]^ or a cobalt(II) macrocyclic complex with a heteroleptic copper(I) complex,^[17]^ while more recently notable results were also achieved in aqueous solution using a cobalt porphyrin as a catalyst and a water‐soluble organic sensitizer.^[18]^

**Scheme 1:**
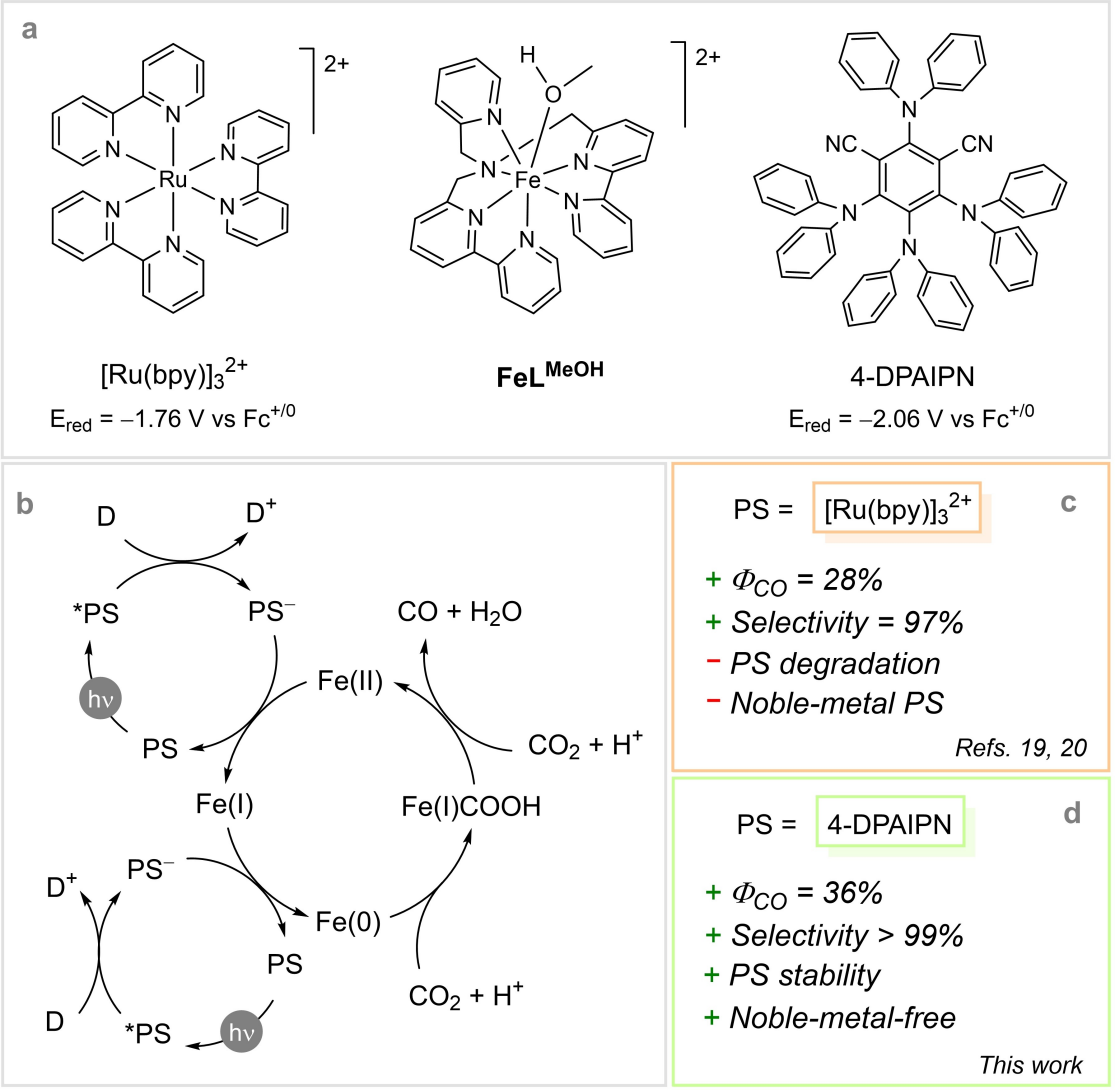
a) Molecular structures of the compounds discussed in the present work, b) mechanism of light‐driven catalysis of the CO_2_RR to CO by **FeL^MeOH^** and relevant key performance indicators using c) [Ru(bpy)_3_]^2+^ or d) 4‐DPAIPN as the photosensitizer (PS).

We have recently reported that the iron(II) complex **FeL^MeOH^** (Scheme 1) featuring the hexadentate DBPy‐PyA ligand, where DBPy‐PyA=(1‐([2,2’‐bipyridin]‐6‐yl)‐*N*‐([2,2’‐bipyridin]‐6‐ylmethyl)‐*N*‐(pyridin‐2‐ylmethyl) methanamine, acts as an efficient and selective catalyst for the CO_2_RR to CO.[^19^, ^20^] CO formation occurs through an EECC mechanism (where E and C are an electron transfer and a chemical step, respectively) involving CO_2_ coordination at a formal Fe(0) intermediate. Key functional characteristics of the metal complex are: i) a redox‐active ligand able to decrease the basicity of the metal upon reduction thereby minimizing the hydride pathway, ii) a substantial structural flexibility, with the ligand assisting the formation of the metallacarboxylic acid intermediate through intramolecular routes, and iii) a fast catalytic turnover mainly associated with a rapid decarbonylation step.^[20]^ Application of complex **FeL^MeOH^** in combination with [Ru(bpy)_3_]^2+^ (where bpy=2,2’‐bipyridine) as the sensitizer and *N*,*N*‐diisopropylethylamine (DIPEA) as the sacrificial electron donor in acetonitrile, in the presence of 1 M 2,2,2‐trifluoroethanol (TFE) as the proton donor, finally delivered an effective photochemical system allowing conversion of CO_2_ into CO with a quantum yield of up to 28 % and a selectivity of 97 %.^[20]^ For this system, light‐driven catalysis is mainly limited by degradation of the sensitizer, expected to occur due to the slow electron transfer (ET) between the photogenerated [Ru(bpy)_3_]^+^ and the iron complex mainly ascribed to the low exergonicity of the process.

Building upon these findings, we reasoned that increasing the driving force for catalyst activation by the reduced sensitizer could potentially improve the efficiency of light‐driven CO_2_ conversion to CO by **FeL^MeOH^**. In this work, we demonstrate that, by replacing the [Ru(bpy)_3_]^2+^ sensitizer with an organic dye (4‐DPAIPN, Scheme 1), we succeeded in obtaining a noble‐metal‐free photochemical system capable of converting CO_2_ into CO quantitatively with an unprecedented quantum yield of 36 % and a selectivity >99 %. Notably, this novel combination allows to switch the turnover‐limiting process of the photochemical reaction from dye to catalyst degradation, thereby giving full access to the catalytic potential of the **FeL^MeOH^** component.

## Results and Discussion

We started our experimental efforts with the aim of replacing the noble‐metal‐based [Ru(bpy)_3_]^2+^ sensitizer within the three‐component system previously employed for light‐driven CO_2_RR by the **FeL^MeOH^** complex.[^19^, ^20^] With this goal in mind, we targeted a series of thermally‐activated delayed fluorescence (TADF) dyes, namely 4‐CzIPN, 3‐DPAFIPN, and 4‐DPAIPN (Scheme S1).^[21]^ These compounds are indeed able to efficiently absorb visible‐light and their photophysical behavior is associated with a characteristic interplay between the singlet and triplet excited state wherein the small energy gap allows for the occurrence of an efficient reverse intersystem crossing (RISC) enabling the compounds to emit by both prompt (PF) and delayed fluorescence (DF).^[22]^ In this regard, the long (μs) lifetime of the delayed component makes these molecular species promising sensitizers to effectively partake in bimolecular electron transfer processes. Furthermore, they display a reduction power in their one‐electron reduced species that increases in the order 4‐CzIPN < 3‐DPAFIPN < 4‐DPAIPN (Table S1). For all these reasons, these dyes have received considerable interest in the field of photocatalysis[^21^, ^23^, ^24^, ^25^, ^26^, ^27^, ^28^, ^29^] and have been only recently employed for light‐driven CO_2_ reduction in combination with a manganese complex as a catalyst.^[30]^

**Figure 1:**
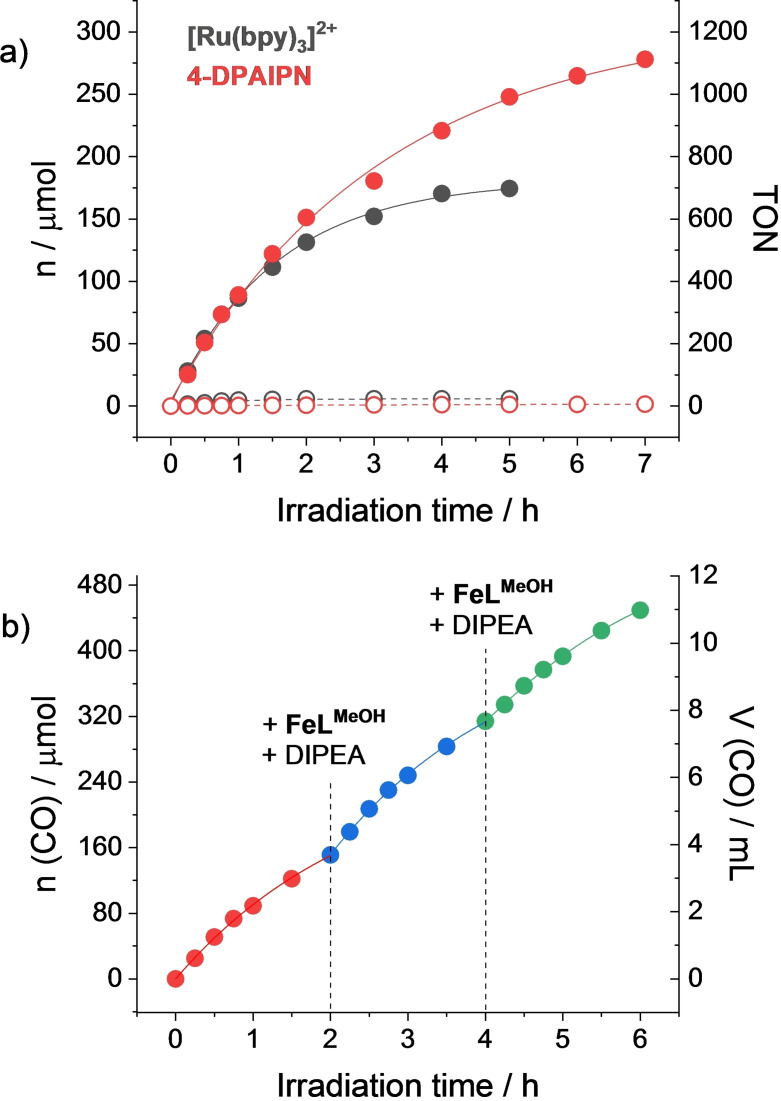
a) Kinetics of product formation (CO: full dots, H_2_: empty dots) upon 1 sun irradiation of CO_2_‐purged acetonitrile solutions containing 0.4 mM sensitizer, 50 μM **FeL^MeOH^**, 0.1 M DIPEA and 1 M TFE; b) kinetics of CO formation upon 1 sun irradiation of CO_2_‐purged acetonitrile solutions containing 0.4 mM 4‐DPAIPN, 50 μM **FeL^MeOH^**, 0.1 M DIPEA and 1 M TFE upon addition of **FeL^MeOH^** and DIPEA after 2 and 4 h.

A preliminary screening of the light‐driven catalytic ability of the **FeL^MeOH^** complex in combination with the TADF dyes (Table S2 and Figure S10) immediately elects 4‐DPAIPN as the most promising noble‐metal free sensitizer, showing catalytic performances even better than the [Ru(bpy)_3_]^2+^ benchmark.^[20]^ Figure 1a shows the comparison of the kinetic traces of CO formation attained using either the [Ru(bpy)_3_]^2+^ or the 4‐DPAIPN sensitizer (0.4 mM in both cases) upon solar‐simulated irradiation (1 sun=0.1 W cm^−2^) of CO_2_‐saturated acetonitrile solutions containing 50 μM **FeL^MeOH^**, 0.1 M DIPEA and 1 M TFE. Remarkably, although CO production starts at similar rates (corresponding to maximum TOFs of 394 and 396 h^−1^ with [Ru(bpy)_3_]^2+^ and 4‐DPAIPN, respectively, it levels off at longer irradiation times in the case of 4‐DPAIPN, leading to a maximum quantity of CO of up to 278 μmol, corresponding to a TON of 1112. Control experiments (Table S3) confirm that CO is solely produced by light‐driven conversion of CO_2_ promoted by the iron complex **FeL^MeOH^**. To the best of our knowledge, the amount of CO here registered represents the largest obtained so far via photochemical means using molecular sensitizers and noble‐metal‐free catalysts (Table S4). Besides, selectivity for CO is practically quantitative using 4‐DPAIPN (>99 % vs. 97 % using [Ru(bpy)_3_]^2+^), still highlighting the benefits attained from the replacement of the ruthenium chromophore. In this regard, differently from what observed in the case of [Ru(bpy)_3_]^2+^,[^19^, ^20^] formation of formate is negligible using 4‐DPAIPN.

Comparison of the absorption spectra before and after photocatalysis (Figure S11) shows that, differently from what observed using the remaining TADF dyes (Figure S12 and S13), the absorption bands of the 4‐DPAIPN dye remain practically unchanged, indicating that degradation of the light‐harvesting component does not represent the limiting factor of the corresponding photocatalytic system. Starting from this evidence, we next examined the turnover limiting factors by performing additions of single components after 2 h of irradiation. Partial recovery of the activity can be attained upon addition of the DIPEA donor (Figure S14), consistent with the high activity of the photochemical system since one molecule of donor is consumed per molecule of CO produced. However, only the simultaneous addition of both the DIPEA donor and the **FeL^MeOH^** catalyst allows to completely restore the production of CO at comparable rates as those measured at the beginning of the experiment (Figure S14). This evidence clearly suggests that catalyst degradation represents the main limiting factor using 4‐DPAIPN as the photosensitizer. These results differ substantially from those obtained using [Ru(bpy)_3_]^2+^ for which the levelling off of the light‐driven activity was associated with degradation of the light‐harvesting component.^[20]^ Thus, the replacement of the [Ru(bpy)_3_]^2+^ chromophore with the 4‐DPAIPN dye also enables to drastically switch the turnover limiting process from dye to catalyst degradation. Building upon these findings, addition of 50 μM **FeL^MeOH^** and parallel recovery of the initial concentration of DIPEA every 2 hours allowed us to achieve the generation of up to 11 mL of CO from 5 mL of solution after only 6 h (Figure 1b).

**Figure 2:**
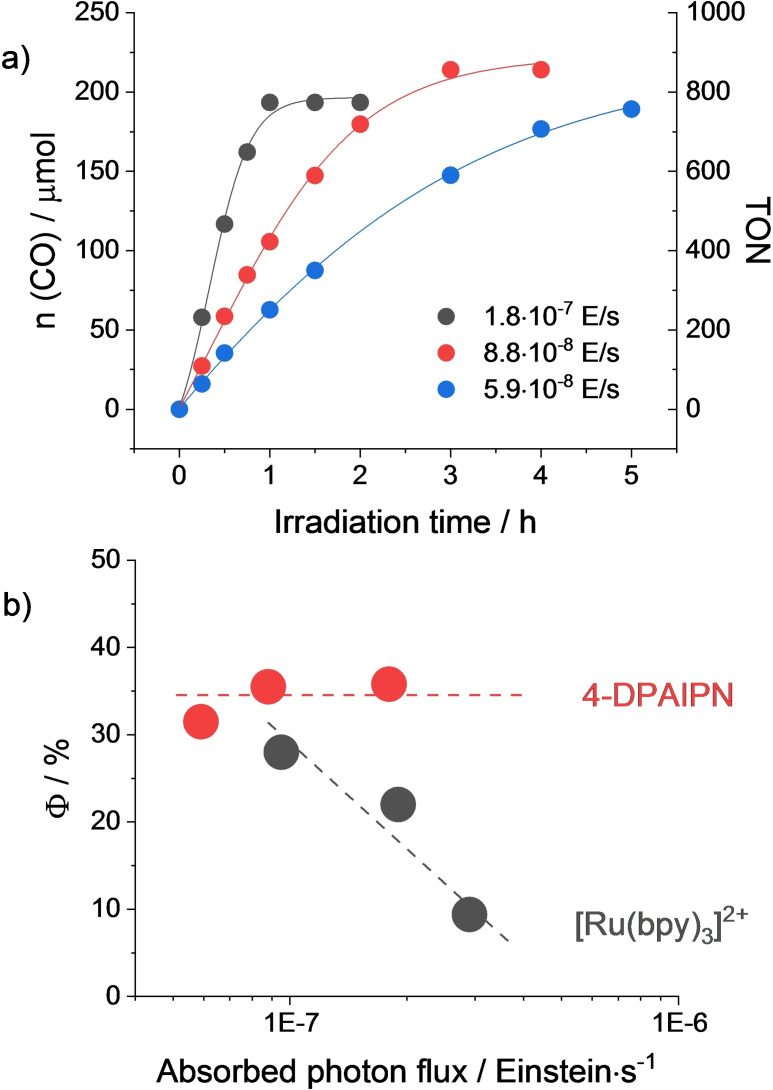
a) Kinetics of CO formation upon irradiation with a 460 nm LED at variable absorbed photon flux of CO_2_‐purged acetonitrile solutions containing 0.4 mM 4‐DPAIPN, 50 μM **FeL^MeOH^**, 0.1 M DIPEA and 1 M TFE; b) trend of the quantum yield of CO formation using either 0.4 mM [Ru(bpy)_3_]^2+^ or 4‐DPAIPN as the light‐harvesting sensitizer.

In order to gain further mechanistic insights, we evaluated the effect of the irradiation power on the light‐driven CO generation using a 460 nm LED (Figure 2). Interestingly, using 4‐DPAIPN as the sensitizer we observed an increase in the rate of CO formation upon increasing the photon flux (Figure 2a), resulting in appreciably constant quantum yields, reaching a record value of Φ=36 % (Figure 2b). The observed trend strongly suggests that CO formation using the 4‐DPAIPN dye is not limited by the photogeneration and fate of the reduced sensitizer, rather it is presumably limited by the dark chemical steps involving the **FeL^MeOH^** complex. Conversely, in the case of the [Ru(bpy)_3_]^2+^ sensitizer, the quantum yield is inversely dependent on the photon flux and the highest value of 28 % is reached only at low irradiation power (Figure 2b).^[20]^ For this latter system, catalyst reduction by the photogenerated [Ru(bpy)_3_]^+^ indeed represents the rate‐determining step of the catalysis, thus the decrease of its concentration at steady‐state allows to optimize the efficiency of the overall process by minimizing its detrimental deactivation pathways.^[31]^

To elucidate the reasons for the observed enhancement in activity attained using the 4‐DPAIPN dye, we performed spectroscopic measurements by merging time‐resolved emission and absorption experiments. Catalyst activation is expected to involve reductive quenching of the excited state of the 4‐DPAIPN dye by the DIPEA, followed by ET from the reduced 4‐DPAIPN radical anion to **FeL^MeOH^** (Scheme 1b).[^30^, ^32^] We thus performed time‐resolved emission analysis by both time‐correlated single‐photon counting (TCSPC) and laser flash photolysis (LFP) to extract mechanistic information on the primary photochemical process. These experiments showed that only the delayed component of the 4‐DPAIPN fluorescence (DF) is quenched by the DIPEA electron donor, consistent with reaction involving the triplet excited state (see SI, Section 4). For this process, Stern‐Volmer analysis (Figure S19) yields a bimolecular rate constant of *k*=6.2×10^5^ M^−1^ s^−1^. This value is ca one‐order of magnitude lower that that measured for the triplet excited state of [Ru(bpy)_3_]^2+^,^[19]^ likely resulting from the lower driving force (by ca 100 mV) for the ET process. Though intrinsically slower, reductive quenching of the triplet of 4‐DPAIPN at 0.1 M DIPEA is however rather efficient (η_q_ ∼70 %) due to the long excited‐state lifetime (τ=34 μs for the DF component in N_2_‐purged acetonitrile, see Figure S3).

**Figure 3:**
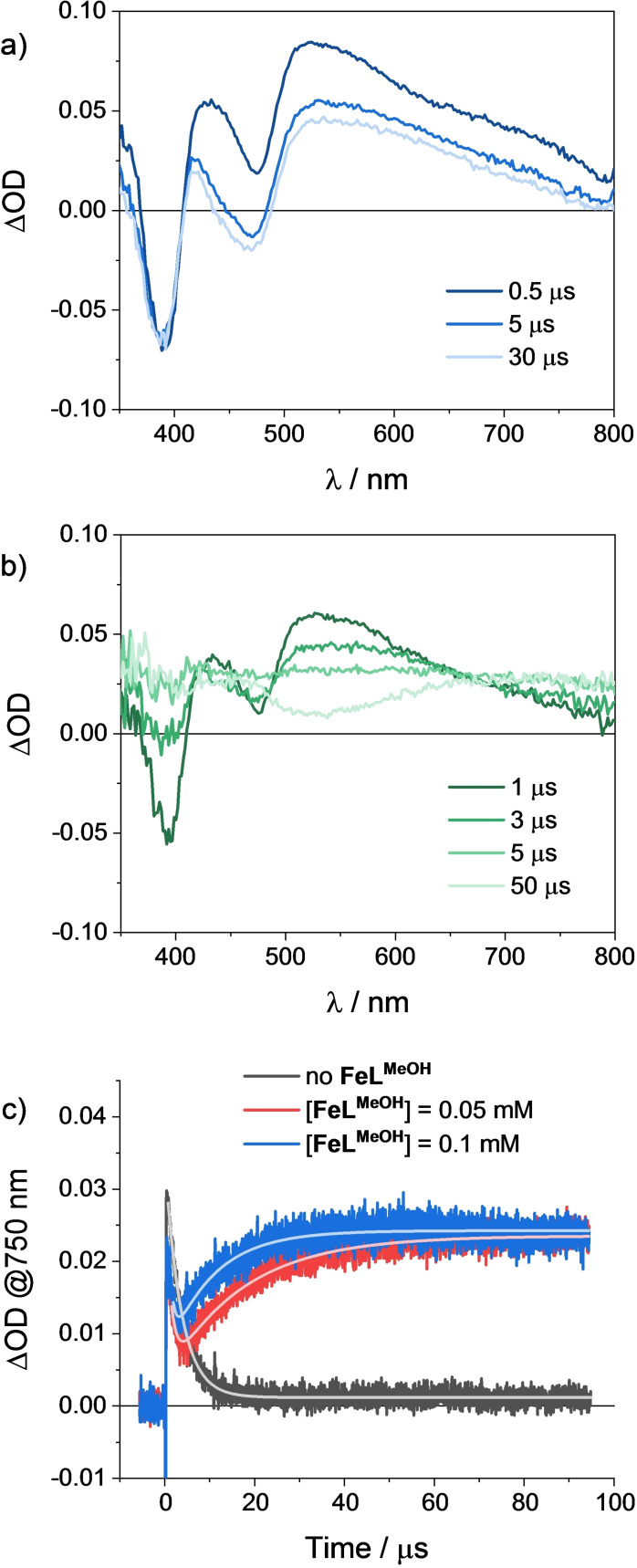
Spectral evolution of an acetonitrile solution containing a) 25 μM 4‐DPAIPN and 0.5 M DIPEA and b) 25 μM 4‐DPAIPN, 0.5 M DIPEA, and 0.25 mM **FeL^MeOH^** obtained by LFP (excitation at 355 nm), c) kinetic analysis at 750 nm with 0, 0.05, and 0.1 mM **FeL^MeOH^**.

The reaction involving the photogenerated 4‐DPAIPN radical anion and the **FeL^MeOH^** catalyst was subsequently followed by transient absorption spectroscopy. The spectral evolution obtained by LFP (excitation at 355 nm) of an acetonitrile solution containing 25 μM 4‐DPAIPN and 0.5 M DIPEA is depicted in Figure 3a. The prompt spectrum shows a broad absorption in the visible with relative maxima at 430 and 520 nm and superimposed bleaching at 380 and 470 nm which can be assigned to the triplet excited state of 4‐DPAIPN. These spectral features subsequently undergo a decrease in ΔOD in the 400–800 nm region, leading to the stable formation of a transient spectrum with similar absorptions, but enhanced bleaching at 470 nm. This latter can be assigned to the 4‐DPAIPN radical anion for comparison with literature reports.[^33^, ^34^] Kinetic analysis at 470 nm (Figure S20) yields a time‐constant of 2.8 μs for this electron transfer process, in good agreement with the lifetime expected for the triplet excited state of 4‐DPAIPN in the presence of 0.5 M DIPEA, based on the Stern‐Volmer analysis previously discussed. The absorption fingerprint of the 4‐DPAIPN radical anion then remains persistent in time (Figure S20), as expected based upon the irreversible nature of the electron transfer process. In the presence of the **FeL^MeOH^** catalyst (Figure 3b), the prompt spectrum clearly resembles the signature of the triplet excited state of the dye as previously observed in the absence of the catalyst (Figure 3a). However, this species progressively evolves to a new spectrum featuring an absorption at 450 nm and a broad one with maximum at 750 nm. This latter is the characteristic fingerprint of the reduced Fe(I) catalyst.[^19^, ^20^] Thus, the LFP experiments indicate that, upon excitation, reduction of the catalyst occurs by the photogenerated 4‐DPAIPN radical anion. In this regard, the failure to observe the formation of the reduced dye as an intermediate reflects the similar kinetics of both the reductive quenching of the dye by the DIPEA donor and catalyst reduction by the photogenerated reduced chromophore under these experimental conditions. Kinetic analysis of the formation of the Fe(I) species at 750 nm (Figure 3c) and 470 nm (Figure S21) was finally monitored in the presence of variable concentrations of **FeL^MeOH^** to extract the rate constant of the bimolecular electron transfer from the reduced 4‐DPAIPN to the Fe(II) catalyst. The resulting fitting, performed under pseudo‐first order kinetic conditions (see Section 4 of the SI for details), allows to estimate a *k*=9.2×10^8^ M^−1^ s^−1^ for this reaction (Figure S22). This value is larger by more than one‐order of magnitude than that measured for the photogenerated [Ru(bpy)_3_]^+^,^[20]^ likely resulting from the higher driving force in the case of the 4‐DPAIPN dye, as previously envisioned. More importantly, these results definitely support a fast reactivity between the photogenerated dye and the catalyst, which is instrumental for efficiently feeding the catalytic routine towards the conversion of CO_2_ into CO, while preserving the reduced 4‐DPAIPN from parallel, undesired reaction pathways.

## Conclusions

In conclusion, we have demonstrated that, by coupling an organic TADF dye with a polypyridine iron(II) complex, we have obtained a noble‐metal‐free photochemical system capable of selective CO_2_ reduction to CO with unprecedented performances in terms of amount of product formed and quantum yield. This has been achieved thanks to the fast and efficient electron transfer rates that leads to the activation of the catalyst towards substrate conversion. The fast kinetics is indeed essential to speed up the CO_2_RR catalysis by the iron complex and to preserve the sensitizer from unwanted decomposition pathways, instrumental to enhance the stability of the whole photosystem. Both these evidences clearly point out the privileged role of organic TADF photosensitizers when compared to common metal‐based chromophores such as the well‐known [Ru(bpy)_3_]^2+^. Furthermore, these results highlight the great potential of molecular species based on cheap and abundant elements to target relevant catalytic transformations in the context of solar fuel formation. We believe that the possibility of generating CO efficiently and quantitatively from small reaction volumes through photochemical means will open new avenues in the exploitation of CO_2_ as a surrogate for carbonylation reactions.[^35^, ^36^, ^37^]

## Experimental Section

All reagents were obtained from standard suppliers and used without additional purification. Complex **FeL^MeOH^** was synthesized following literature protocols.^[19]^ 4‐DPAIPN, 3‐DPAFIPN, and 4‐CzIPN were prepared according to reported procedures.^[38]^ Absorption spectra were recorded at room temperature using an Agilent Technologies spectrophotometer. Luminescence spectra were recorded using an Edinburgh Instrument spectrofluorometer. Fluorescence lifetimes were measured using a TCSPC apparatus (PicoQuant Picoharp 300) equipped with a subnanosecond LED source at 380 nm (500–700 ps pulse width) powered by a PicoQuant PDL 800‐B variable (2.5–40 MHz) pulsed power supply. The decays were analyzed by means of PicoQuant FluoFit Global Fluorescence Decay Analysis Software. Transient absorption spectroscopy measurements were conducted using a custom laser spectrometer comprising of a Continuum Surelite II Nd: YAG laser (FWHM=8 ns) with frequency tripled option (355 nm). Photomultiplier signals (kinetic traces) were processed using a Teledyne LeCroy 604Zi (400 MHz, 20 GS/s) digital oscilloscope. Transient spectra were recorded using a PiMax CCD Camera. Light‐driven CO_2_RR experiments were performed under continuous visible light irradiation using two different light sources: a 3 W 460 nm LED (spectrum reported in Figure S1) and a 175 W Xenon Arc‐lamp (CERMAX PE175BFA). For the latter, an incident power of 1 sun (0.1 W cm^−2^) was set using a power meter. In a typical photochemical experiment, samples were prepared in 20 mL scintillation vials by mixing stock solutions of the dye and the catalyst, followed by the addition of DIPEA and TFE. The solution was then placed in the reactor, purged with CO_2_ for 20 minutes, continuously stirred, and maintained at a constant temperature of 15 °C. Experiments were run in duplicate and the results are reported as the average of two independent experiments. The measuring cell is sealed during the reaction: the head to which the cell is attached has four ports, closed with Swagelok^®^ connections, two of them are part of a closed loop involving GC gas inlet and sample vent to analyze the headspace content without an appreciable gas consumption, and the other two are for the degassing procedure (input and output). The gas phase of the reaction vessel was analyzed on an Agilent Technologies 490 micro‐GC equipped with a 5 Å molecular sieve column (10 m), a thermal conductivity detector, and using Ar as the carrier. The unused gas sample is then reintroduced in the reactor to minimize its consumption along the whole photolysis. The moles of gases were quantified through the external calibration method. In the case of H_2_, this procedure was performed through a galvanostatic (typically 1 mA) electrolysis of a 0.1 M H_2_SO_4_ solution. A 100 % faradaic efficiency was assumed leading to a linear correlation between the amount of H_2_ evolved at the cathode and the electrolysis time. CO was quantified using a response factor obtained by injecting known amounts of gas in the cell and then sampling the headspace. Average errors are within ±10 % for CO and ±5 % for H_2_. The presence of formate was checked using ^1^H‐NMR (δ=8.3 ppm) following literature protocols.^[19]^ The photolyzed solution was brought to basic pH upon the addition of the minimum amount of NaOH and then evaporated under reduced pressure. The solid residue was then dissolved in 2 mL D_2_O and sonicated for 5 min, dimethylformamide (1 μL, δ=7.8 ppm) was then added as an internal standard and the resulting solution was filtered before entering the NMR tube.

## Conflict of Interests

The authors declare no conflict of interest.

1

## Supporting information

As a service to our authors and readers, this journal provides supporting information supplied by the authors. Such materials are peer reviewed and may be re‐organized for online delivery, but are not copy‐edited or typeset. Technical support issues arising from supporting information (other than missing files) should be addressed to the authors.

Supporting Information

## Data Availability

The data that support the findings of this study are available from the corresponding author upon reasonable request.

## References

[1] A. Morris , G. J. Meyer , E. Fujita , Acc. Chem. Rev. 2009, 42, 1983–1994. 10.1021/ar9001679 19928829

[2] E. E. Benson , C. P. Kubiak , A. Sathrum , J. M. Smieja , Chem. Soc. Rev. 2009, 38, 89–99. 19088968 10.1039/b804323j

[3] A. Nakada , H. Kumagai , M. Robert , O. Ishitani , K. Maeda , Acc. Mater. Res. 2021, 2, 458–470.

[4] Y. Matsubara , D. Grills , Y. Kuwahara , ACS Catal. 2015, 11, 6440–6452.

[5] M. L. Pegis , J. A. S. Roberts , D. J. Wasylenko , E. A. Mader , A. M. Appel , J. M. Mayer , Inorg. Chem. 2015, 54, 11883–11888. 26640971 10.1021/acs.inorgchem.5b02136

[6] I. Azcarate , C. Constentin , M. Robert , J.-M. Savéant , J. Am. Chem. Soc. 2016, 138, 16639–16644. 27976580 10.1021/jacs.6b07014

[7] S. Gonell , J. Lloret-Fillol , A. J. M. Miller , ACS Catal. 2021, 11, 615–626.

[8] M. Bourrez , F. Molton , S. Cardon-Noblat , A. Deronzier , Angew. Chem. Int. Ed. 2011, 50, 9903–9906. 10.1002/anie.201103616 21922614

[9] J.-S. Derrick , M. Loipersberger , R. Chatterjee , D. A. Iovan , P. T. Smith , K. Chakarawet , J. Yano , J. R. Long , M. Head-Gordon , C. J. Chang , J. Am. Chem. Soc. 2020, 142, 20489–20501. 33207117 10.1021/jacs.0c10664

[10] F. Droghetti , A. Amati , A. Ruggi , M. Natali , Chem. Commun. 2024, 60, 658–673. 10.1039/d3cc05156k 38117176

[11] H. Takeda , K. Koike , H. Inoue , O. Ishitani , J. Am. Chem. Soc. 2008, 130, 2023–2031. 18205359 10.1021/ja077752e

[12] R. Kuriki , H. Matsunaga , T. Nakashima , K. Wada , A. Yamakata , O. Ishitani , K. Maeda , J. Am. Chem. Soc. 2016, 138, 5159–5170. 27027822 10.1021/jacs.6b01997

[13] P. De La Torre , J. S. Derrick , A. Snider , P. T. Smith , M. Loipersberger , M. Head-Gordon , C. J. Chang , ACS Catal. 2022, 12, 8484–8493.

[14] L. L. Gracia , E. Barani , J. Braun , A. B. Carter , O. Fuhr , A. K. Powell , K. Fink , C. Bizzarri , ChemCatChem 2022, 14, e202201163.

[15] Y. Xue , D. Chao , ChemPhotoChem 2024, 8, e202400246.

[16] P. Y. Ho , S. C. Cheng , F. Yu , Y. Y. Yeung , W. X. Ni , C. C. Ko , C. F. Leung , T. C. Lau , M. Robert , ACS Catal. 2023, 13, 5979–5985.

[17] J. W. Wang , Z. Li , Z. M. Luo , Y. Huang , F. Ma , S. Kupfer , G. Ouyang , Proc. Natl. Acad. Sci. U. S. A. 2023, 120, e2221219120. 36943881 10.1073/pnas.2221219120 PMC10068849

[18] F. Ma , Z. M. Luo , J. W. Wang , G. Ouyang , J. Am. Chem. Soc. 2024, 146, 17773–17783. 38888951 10.1021/jacs.4c03128

[19] F. Droghetti , A. Amati , F. Pascale , A. Crochet , M. Pastore , A. Ruggi , M. Natali , ChemSusChem 2024, 22, e202300737. 10.1002/cssc.202300737 37846888

[20] F. Droghetti , F. Lemken , L. Rulisek , A. Ruggi , M. Natali , ACS Catal. 2024, 14, 16920–16935.

[21] A. Vega-Peñaloza , J. Mateos , X. Companyó , M. Escudero-Casao , L. Dell'Amico , Angew. Chem. Int. Ed. 2021, 133, 1096–1111. 10.1002/anie.202006416 32568437

[22] M. Y. Wong , E. Zysman-Colman , Adv. Mater. 2017, 29, 1605444. 10.1002/adma.201605444 28256751

[23] T. Y. Shang , L. H. Lu , Z. Cao , Y. Liu , W. M. He , B. Yu , Chem. Commun. 2019, 55, 5408–5419. 10.1039/c9cc01047e 31020957

[24] P. Franceschi , E. Rossin , G. Goti , A. Scopano , A. Vega-Peñaloza , M. Natali , D. Singh , A. Sartorel , L. Dell'Amico , J. Org. Chem. 2023, 88, 6454–6464. 36760023 10.1021/acs.joc.2c02952 PMC10204093

[25] Q. Y. Meng , T. E. Schimer , A. L. Berger , K. Donabauer , B. König , J. Am. Chem. Soc. 2019, 141, 11393–11397. 31280561 10.1021/jacs.9b05360 PMC6686948

[26] R. I. Rodriguez , V. Corti , L. Rizzo , S. Visentini , M. Bortolus , A. Amati , M. Natali , G. Pelosi , P. Costa , L. Dell'Amico , Nat. Catal. 2024, 7, 1223–1231.

[27] J. Luo , J. Zhang , ACS Catal. 2016, 6, 873–877.

[28] E. Speckmeier , T. G. Fischer , K. Zeitler , J. Am. Chem. Soc. 2018, 140, 15353–15365. 30277767 10.1021/jacs.8b08933

[29] P. P. Singh , V. Srivastava , Org. Biomol. Chem. 2021, 19, 313–321. 33305768 10.1039/d0ob01884h

[30] E. Bassan , R. Inoue , D. Fabry , F. Calogero , S. Potenti , A. Gualandi , P. G. Cozzi , K. Kamogawa , P. Ceroni , Y. Tamaki , O. Ishitani , Sustain. Energy Fuels 2023, 7, 3454–3463.

[31] C. V. Krishnan , N. Sutin , J. Am. Chem. Soc. 1981, 103, 2141–2142.

[32] F. Droghetti , F. Lucarini , A. Molinari , A. Ruggi , M. Natali , Dalton Trans. 2022, 51, 10658–10673. 35475511 10.1039/d2dt00476c PMC9936794

[33] Y. Kwon , J. Lee , Y. Noh , D. Kim , Y. Lee , C. Yu , J. C. Roldao , S. Feng , J. Gierschner , R. Wannemacher , M. S. Kwon , Nat. Commun. 2023, 14, 92. 36609499 10.1038/s41467-022-35774-5 PMC9822901

[34] M. Villa , A. Fermi , F. Calogero , X. Wu , A. Gualandi , P. G. Cozzi , A. Troisi , B. Ventura , P. Ceroni , Chem. Sci. 2024, 15, 14739–14745. 39176242 10.1039/d4sc04518a PMC11337074

[35] P. Gotico , A. Del Vecchio , D. Audisio , A. Quaranta , Z. Halime , W. Leibl , A. Aukauloo , ChemPhotoChem 2018, 2, 715–719.

[36] S. Monticelli , A. Talbot , P. Gotico , F. Caillé , O. Loreau , A. Del Vecchio , A. Malandain , A. Sallustrau , W. Leibl , A. Aukauloo , F. Taran , Z. Halime , D. Audisio , Nat. Commun. 2023, 14, 4451. 37488106 10.1038/s41467-023-40136-w PMC10366225

[37] A. M. Sheta , S. Fernandez , C. Liu , G. C. Dubed-Bandomo , J. Lloret-Fillol , Angew. Chem. Int. Ed. 2024, 63, e202403674. 10.1002/anie.202403674 38647344

[38] S. M. Engle , T. R. Kirkner , C. B. Kelly , Org. Synth. 2019, 96, 455–473.

[39] A. Juris , V. Balzani , F. Barigelletti , S. Campagna , P. Belser , A. Von Zelewsky , Coord. Chem. Rev. 1988, 84, 85–277.

[40] S. P. Pitre , C. D. McTiernan , W. Vine , R. DiPucchio , M. Grenier , J. C. Scaiano , Sci. Rep. 2015, 5, 16397. 26578341 10.1038/srep16397 PMC4649705

[41] Z. Guo , G. Chen , C. Cometto , B. Ma , H. Zhao , T. Groizard , L. Chen , H. Fan , W. L. Man , S. M. Yiu , K. C. Lau , T. C. Lau , M. Robert , Nat. Catal. 2019, 2, 801–808.

