# Supplementary material for: Boosting Light‐Driven CO2 Conversion Into CO by a Polypyridine Iron(II) Catalyst Using an Organic Sensitizer
Source: ChemSusChem. 2025 Feb 6;18(11):e202402627. doi: 10.1002/cssc.202402627 (PMC12131667; doi:10.1002/cssc.202402627)
Supplement: Supplementary file 1 — Supporting Information [file CSSC-18-e202402627-s001.pdf]

ChemSusChem

Supporting Information

Boosting Light-Driven CO_2 Conversion Into CO by a Polypyridine Iron(II) Catalyst Using an Organic Sensitizer

Federico Droghetti, Lucrezia Villa, Andrea Sartorel, Luca Dell'Amico, Albert Ruggi,* and Mirco Natali*

Table of content

1. Methodology p. S2

2. Photophysical and electrochemical properties p. S3

3. Light-driven CO_2RR p. S9

4. Photophysical studies p. S16

5. References of the SI p. S23

1. Methodology

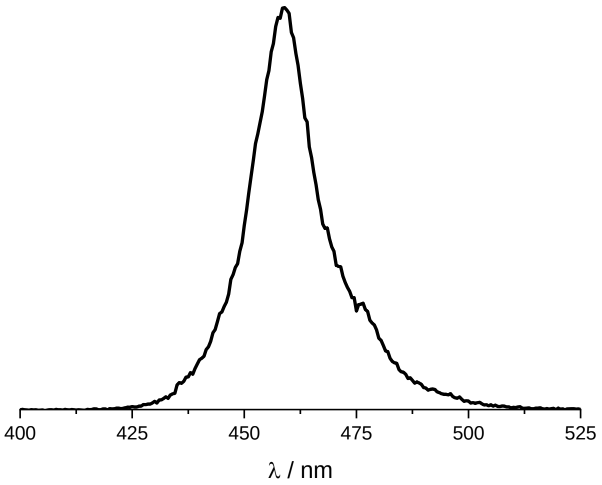

Figure S1. Spectrum of the 460-nm LED employed in the photochemical studies.

2. Photophysical and electrochemical properties

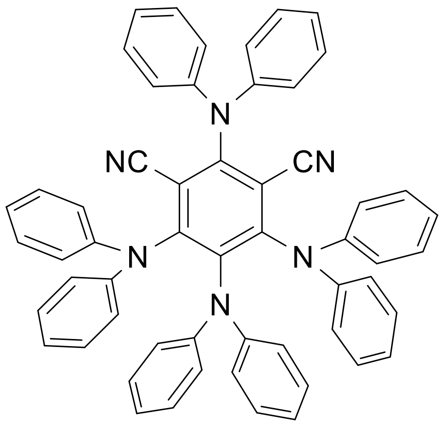

4-DPAIPN

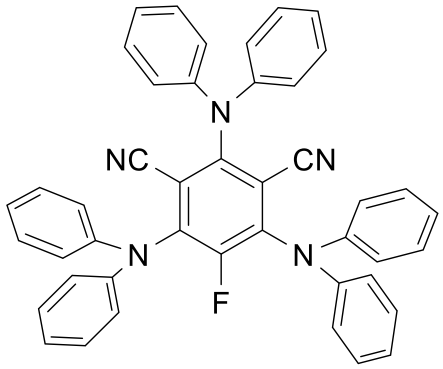

3-DPAFIPN

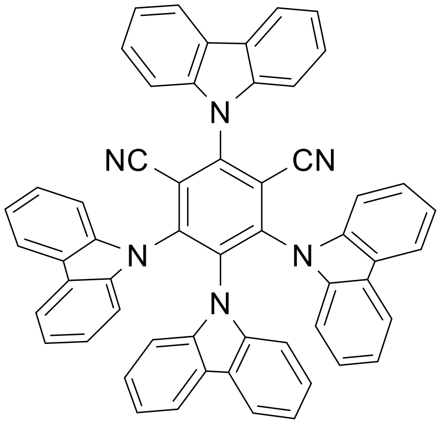

4-CzIPN

Scheme S1. Molecular structures of the TADF dyes investigated in this work.

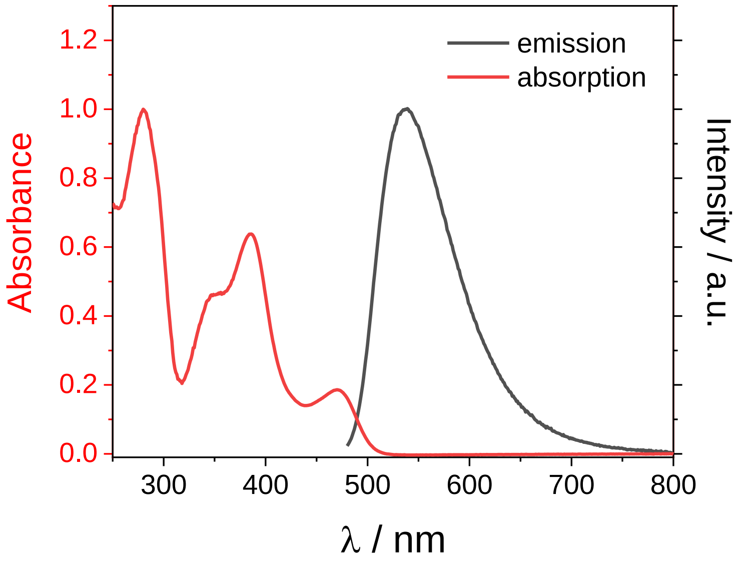

Figure S2. Absorption and emission spectra of 4-DPAIPN in acetonitrile.

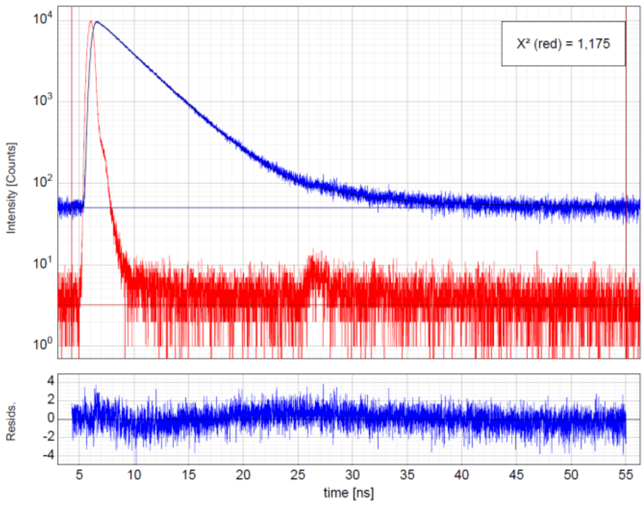

0.

Figure S3. Decay of the prompt fluorescence of 4-DPAIPN in acetonitrile measured by TC-SPC (excitation at 380 nm, analysis at 540 nm). Top panel: decay and monoexponential fitting (blue trace), IRF (red trace); bottom panel: residual curve. A lifetime of 3.40 ns is estimated.

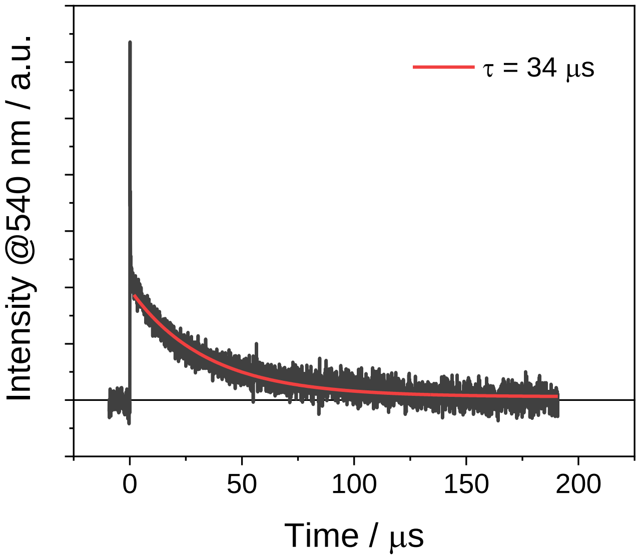

Figure S3. Decay of the delayed fluorescence of 4-DPAIPN in N_2-purged acetonitrile measured by laser flash photolysis (excitation at 355 nm).

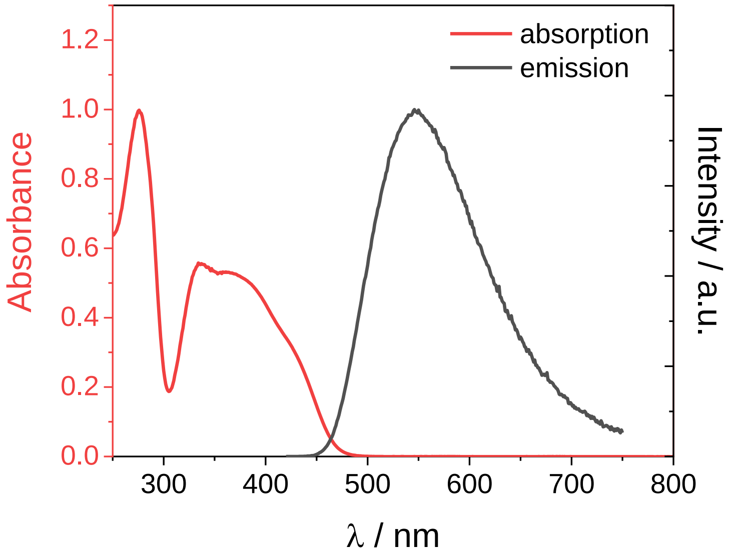

Figure S4. Absorption and emission spectra of 3-DPAFIPN in acetonitrile.

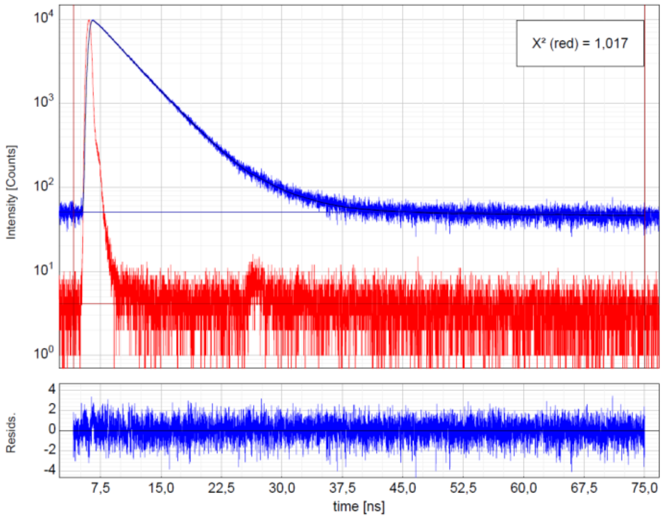

Figure S5. Decay of the prompt fluorescence of 3-DPAFIPN in acetonitrile measured by TC-SPC (excitation at 380 nm, analysis at 550 nm). Top panel: decay and monoexponential fitting (blue trace), IRF (red trace); bottom panel: residual curve. A lifetime of 4.1 ns is estimated.

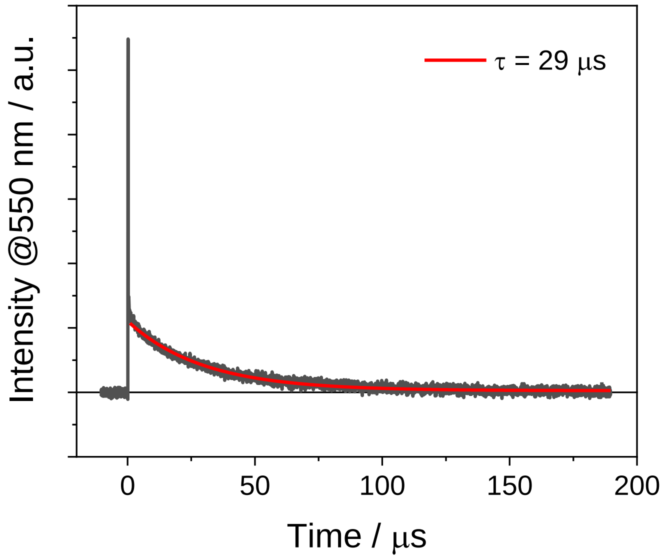

Figure S6. Decay of the delayed fluorescence of 3-DPAFIPN in N_2-purged acetonitrile measured by laser flash photolysis (excitation at 355 nm).

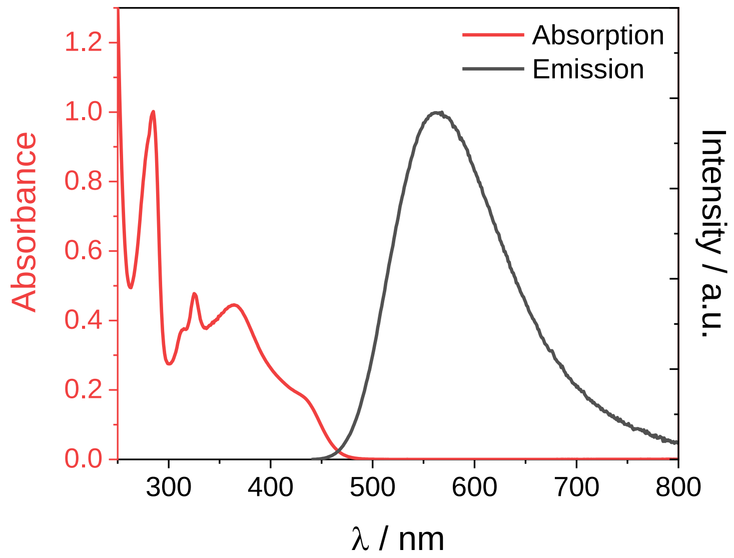

Figure S7. Absorption and emission spectra of 4-CzIPN in acetonitrile.

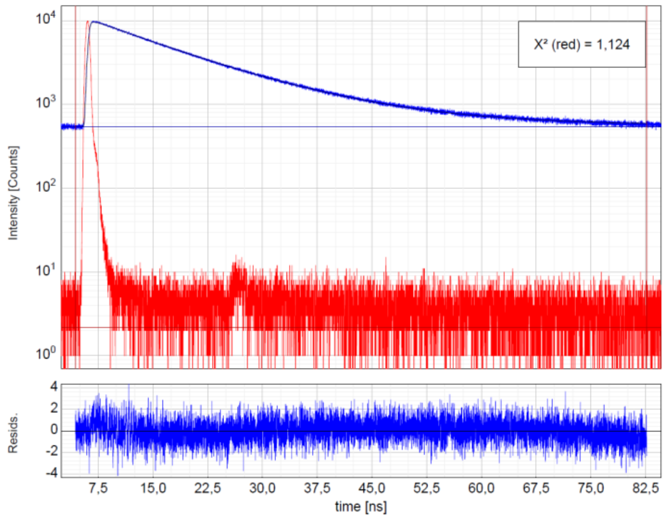

Figure S8. Decay of the prompt fluorescence of 4-CzIPN acetonitrile measured by TC-SPC (excitation at 380 nm, analysis at 550 nm). Top panel: decay and monoexponential fitting (blue trace), IRF (red trace); bottom panel: residual curve. A lifetime of 12.9 ns is estimated.

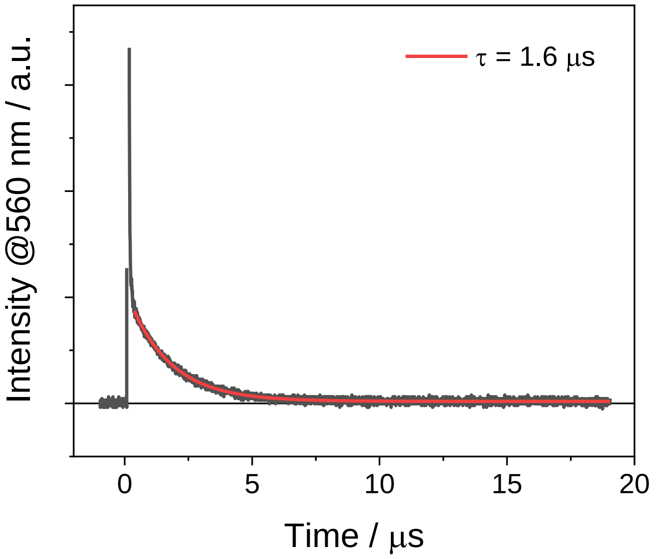

Figure S9. Decay of the delayed fluorescence of 4-CzIPN in N_2-purged acetonitrile measured by laser flash photolysis (excitation at 355 nm).

Table S1. Comparison of the electrochemical properties in acetonitrile of $[Ru(bpy)_3]^{2+}$ and the TADF dyes tested in the present work, potentials are all referenced to Fc^+/Fc.[S1,S2]

	E_{PS/PS^-} / V	$E_{PS^+/PS}$ / V	$E_{PS^+/*PS}$ / V	E_{*PS/PS^-} / V
**$[Ru(bpy)_3]^{2+}$**	−1.76	+0.85	−1.25	+0.34
**4-DPAIPN**	−2.06	+0.61	−1.69	+0.24
**3-DPAFIPN**	−1.99	+0.91	−1.78	+0.69
**4-CzIPN**	−1.64	+1.09	−1.58	+1.03

3. Light-driven CO_2RR

The photochemical activity was evaluated by considering diverse key performance indicators that can be extrapolated from the kinetics traces:

1) The total amount of products (CO, H_2) estimated at the plateau of the kinetics.

2) The maximum turnover number (TON) is estimated by eq. S1, where the n_{max} and n_{cat} are the maximum moles of product and the moles of the catalyst, respectively.

$$TON = \frac{n_{max}}{n_{cat}} \qquad \text{(S1)}$$

3) The initial rate of product (CO and H_2) formation (r, mol·s^{-1}) calculated from the slope in the linear portion of the kinetic trace.

4) The maximum turnover frequency (TOF, s^{-1}) defined according to eq. S2.

$$TOF = \frac{r}{n_{cat}} \qquad \text{(S2)}$$

5) The quantum yield (Φ) calculated according to eq. S3, where φ is the absorbed photon flux (Einstein·s^{-1}) estimated via actinometry.

$$\Phi = \frac{r}{\varphi} \qquad \text{(S3)}$$

The quantification of the absorbed photon flux (φ) under 460-nm LED irradiation was obtained by employing the photoreaction between $[Ru(bpy)_3]Cl_2{\cdot}6H_2O$ and 9,10-diphenylanthracene (DPA) in aerated acetonitrile solution as an actinometer, following an established protocol.[S3] A 6 mL solution consisting of 0.4 mM $[Ru(bpy)_3]Cl_2{\cdot}6H_2O$ and 0.1 mM DPA in acetonitrile was prepared and its UV-Vis spectrum was recorded. Subsequently, the solution was subjected to a one-minute irradiation

using the same light source and setup employed in the photochemical experiments. A new UV-Vis spectrum of the irradiated sample was then acquired. Both samples were diluted for the UV-Vis spectra measurements by mixing 1 mL of the sample with 2 mL of acetonitrile. The moles of DPA consumed were computed following eq. S4 where A_i is the absorbance at 372 nm before the irradiation, A_f is the absorbance at 372 nm after the irradiation, V is the volume of the sample (considering dilution) and ε_{372nm} = 11,100 $M^{-1}cm^{-1}$ is the attenuation coefficient of DPA at 372 nm in acetonitrile. A cuvette with a 1 cm path length was employed.

$$n\ DPA\ consumed = \frac{A_i - A_f}{\varepsilon_{372\ nm}} \times V \quad \text{(S4)}$$

The absorbed photon flux (φ) was calculated using eq. S5 where Φ_{ref} = 0.019 is the quantum yield of the actinometer and t = 60 s is the irradiation time.

$$\varphi = \frac{n\ DPA\ consumed}{\Phi_{ref}\ t} \quad \text{(S5)}$$

Table S2. Comparison of the relevant key performance indicators of light-driven CO_2RR for the photosensitizers (PSs) discussed in the present work.[a]

		**n / µmol (TON)**		**Selectivity / %** [b]		
**Entry**	**PS**	**H_2**	**CO**	**H_2**	**CO**	**TOF_{CO} / h^{-1}**
1	4-DPAIPN	1.5 (6)	278.0 (1112)	0.5	99.5	396
2	4-CzIPN	1.6 (6)	127.0 (508)	1	99	89
3	3-DPAFIPN	3.6 (14)	11.5 (46)	23	77	- [c]
4	$[Ru(bpy)_3]^{2+}$	5.8 (23)	174.5 (698)	3	97	394

[a] Irradiation with Xe lamp (1 sun = 0.1 W·cm^{-2}), 0.1 M DIPEA, 0.4 mM PS, 1 M TFE, 50 µM **FeL^{MeOH}** in acetonitrile after 20 min bubbling of CO_2; [b] estimated as the ratio between the amount of a single product and the total amount of products; [c] not determined due to rapid abatement of the catalytic activity.

Table S3. Control experiments.[a]

	**n / µmol** [g]		
**Entry**	**H_2**	**CO**	**Formate**
1 [b]	-	-	-
2 [c]	-	-	-
3 [d]	-	-	-
4 [e]	0.7	1.4	-
5 [f]	1.0	-	-

[a] Xe lamp (1 sun = 0.1 W·cm^{-2}), 0.1 M DIPEA, 0.4 mM 4-DPAIPN, 1 M TFE, 50 µM **FeL^{MeOH}** in acetonitrile after 20 min bubbling of CO_2; [b] no light; [c] no DIPEA; [d] no 4-DPAIPN; [e] no **FeL^{MeOH}**; [f] degassed using Ar instead of CO_2; [g] estimated after 5 h of irradiation.

Table S4. Comparison of the most productive photosystems based on noble-metal-free catalysts for light-driven CO_2RR to CO reported up to date (a single run is herein considered).

Cat / PS / D / Solvent	$n_{CO} \cdot V_{solution}^{-1}$ / µmol·mL^{-1}	TON_{CO} ([Cat])	CO / %	Irradiation time / h (light)	Ref
**FeL^{MeOH}**<br>4-DPAIPN<br>DIPEA<br>ACN / TFE	55.6	1112 (50 µM)	99.5	7 (1 sun)	This work
**FeL^{MeOH}**<br>$[Ru(bpy)_3]^{2+}$<br>DIPEA<br>ACN / TFE	37.5	750 (50 µM)	97	5 (1 sun)	[S4]
$[Fe(tpyPY2Me)]^{2+}$<br>$[Ru(bpy)_3]^{2+}$<br>BIH<br>ACN / PhOH	31.6	15520 (2 µM)	99	1.5 (blue LED)	[S5]
$[Co(qpy)(OH_2)_2]^{2+}$<br>$TATA^+$<br>BIH / TEOA<br>8/2 ACN/H_2O	38.0	18989 (2 µM)	93	24 (LED@471nm)	[S6]
$[CoPyN5]^{2+}$<br>$[Cu(xantphos)(pybcp)]^+$<br>BIH / TEA<br>ACN / TFE	33.8	338 (100 µM)	97	2 (LED@425nm)	[S7]
Co(oTMPyP)<br>4P-DPAIPN<br>Ascorbate<br>$NaHCO_3$ buffer	20.9	2100 (5 µM)	93	21 (LED@450nm)	[S8]
$[Co_2(biqpy)]^{4+}$<br>$[Ru(phen)_3]^{2+}$<br>BIH<br>ACN / PhOH	41.4	829 (50 µM)	96	1.5 (LED@460nm)	[S9]

Legend: ACN = acetonitrile; BIH = 1,3-dimethyl-2-phenyl-2,3-dihydro-1H-benzo[d]-imidazole; DIPEA = *N*,*N*-diisopropylethilamine; PhOH = phenol $TATA^+$ = 4,8,12-tri-(n-butyl)triazatriangulenium; TEOA = triethanolamine; TEA = triethylamine; TFE = 2,2,2-trifluoroethanol.

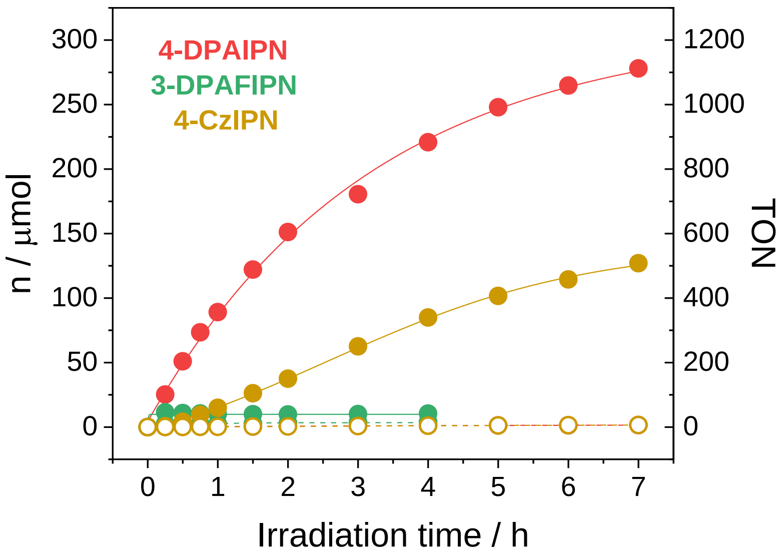

Figure S10. Kinetics of CO (full dots) and H_2 (empty dots) formation upon 1 sun irradiation of CO_2-purged acetonitrile solutions containing 0.4 mM organic sensitizer, 50 µM **FeL^{MeOH}**, 0.1 M DIPEA and 1 M TFE.

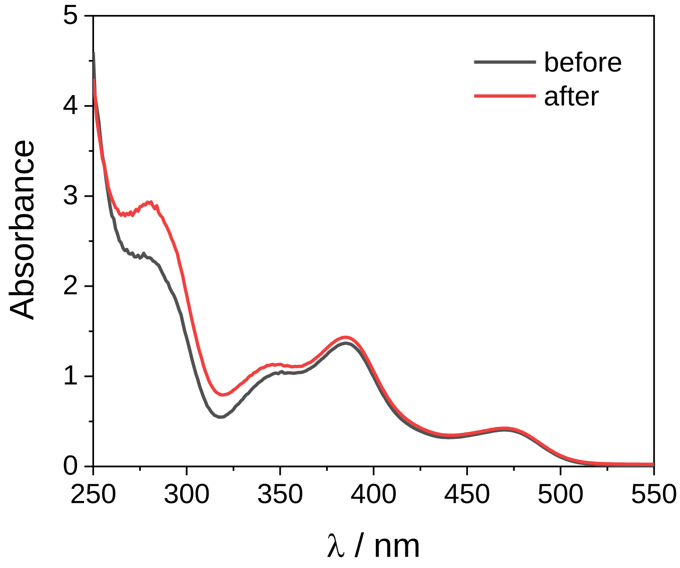

Figure S11. Comparison of the absorption spectra (optical path length = 0.1 cm) before and after 7 h irradiation. Experimental conditions: 1 sun irradiation of CO_2-purged acetonitrile solution containing 0.4 mM 4-DPAIPN, 50 µM **FeL^{MeOH}**, 0.1 M DIPEA and 1 M TFE.

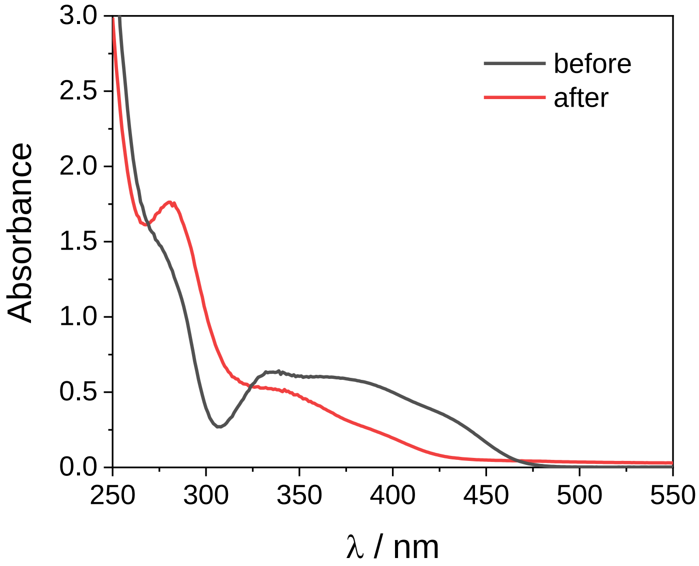

Figure S12. Comparison of the absorption spectra (optical path length = 0.1 cm) before and after 4 h irradiation. Experimental conditions: 1 sun irradiation of CO_2-purged acetonitrile solution containing 0.4 mM 3-DPAFIPN, 50 μM **FeL**$^{\mathbf{MeOH}}$, 0.1 M DIPEA and 1 M TFE.

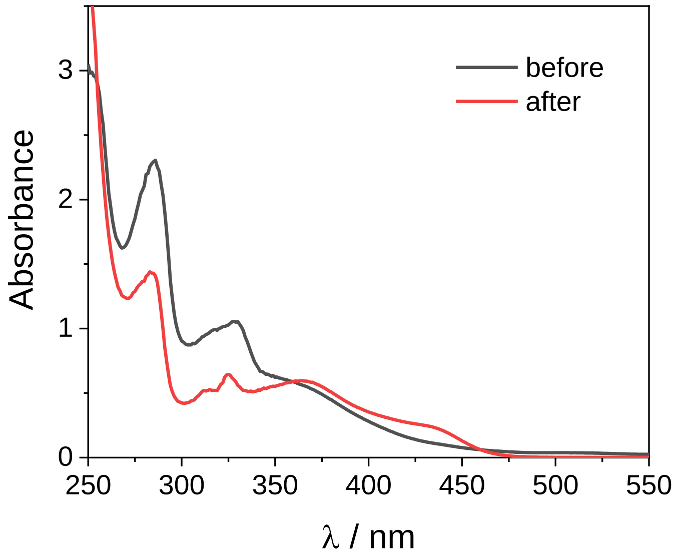

Figure S13. Comparison of the absorption spectra (optical path length = 0.1 cm) before and after 7 h irradiation. Experimental conditions: 1 sun irradiation of CO_2-purged acetonitrile solution containing 0.4 mM 4-CzIPN, 50 μM **FeL**$^{\mathbf{MeOH}}$, 0.1 M DIPEA and 1 M TFE.

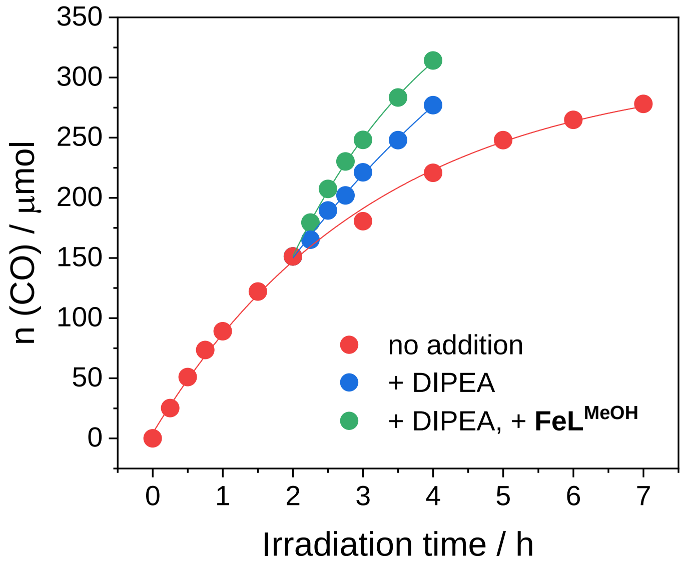

Figure S14. Resumption of the light-driven catalytic activity after 2 h upon addition of 150 µmol DIPEA (blue) and 150 µmol DIPEA + 50 µM **FeL**$^{\mathbf{MeOH}}$ (green). Initial conditions: 1 sun, CO_2-purged acetonitrile, 0.4 mM 4-DPAIPN, 50 µM **FeL**$^{\mathbf{MeOH}}$, 0.1 M DIPEA and 1 M TFE.

4. Photophysical studies

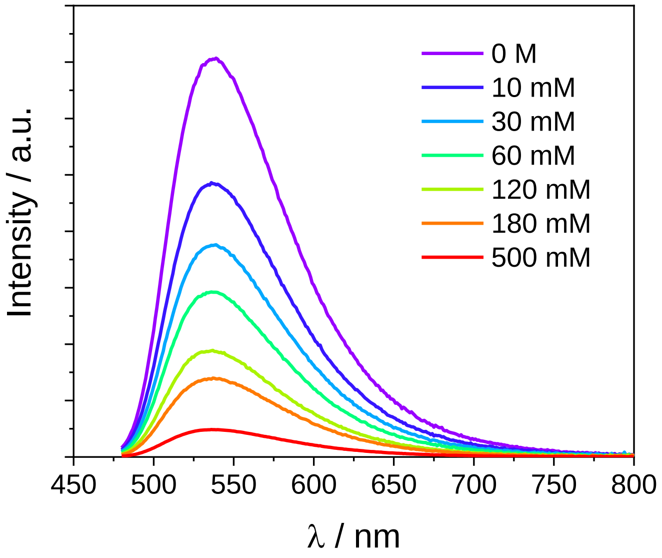

Figure S15. Luminescence spectra of 4-DPAIPN in acetonitrile solution in the presence of variable concentrations of DIPEA.

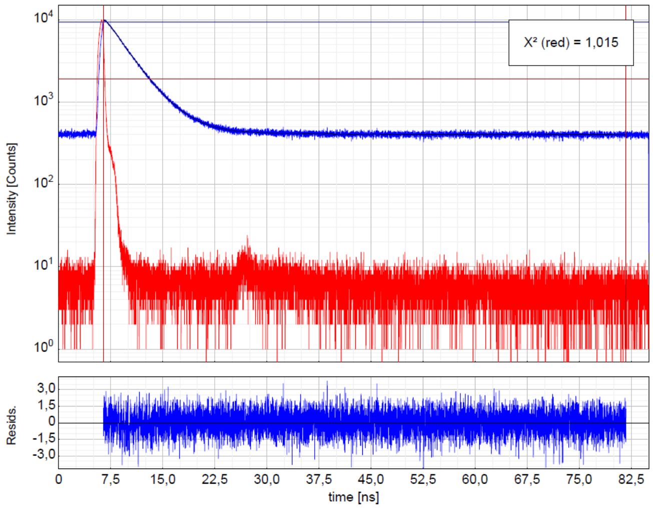

Figure S16. Decay of the prompt fluorescence of 4-DPAIPN in acetonitrile solution in the presence of 180 mM DIPEA measured by TCSPC (excitation at 380 nm, analysis at 540 nm). Top panel: decay and monoexponential fitting (blue trace), IRF (red trace); bottom panel: residuals curve. A lifetime of 3.34 ns is estimated.

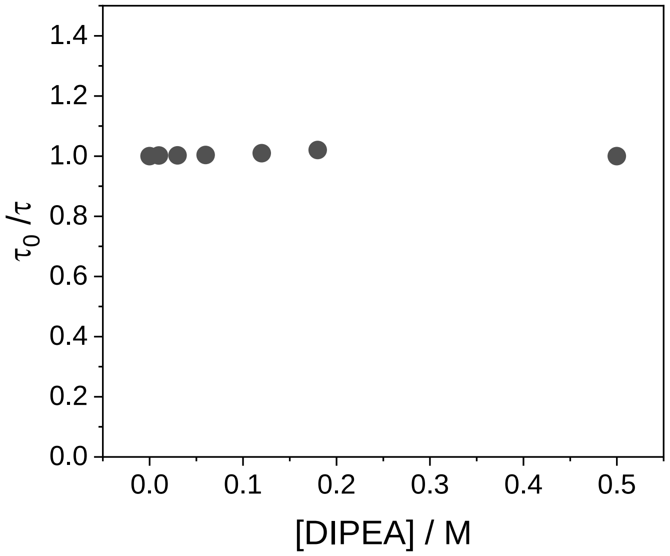

Figure S17. Stern-Volmer analysis of the quenching of the prompt fluorescence of 4-DPAIPN by DIPEA measured by TCSPC (excitation at 380 nm, analysis at 540 nm).

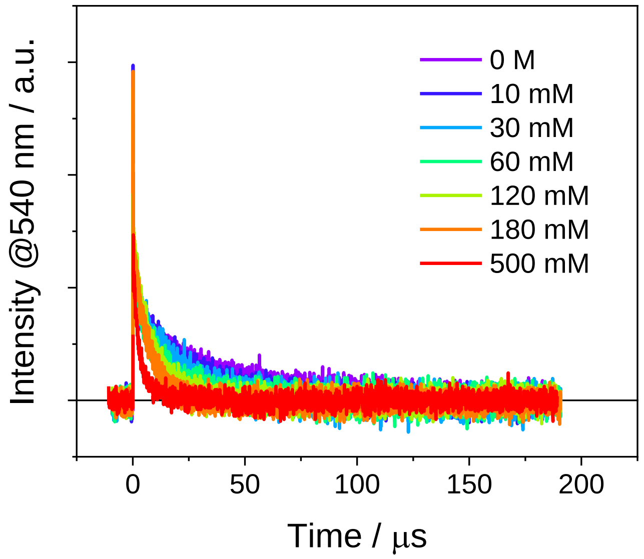

Figure S18. Decays of the delayed fluorescence component of 4-DPAIPN in N_2-purged acetonitrile solution in the presence of variable concentrations of DIPEA measured by laser flash photolysis (excitation at 355 nm).

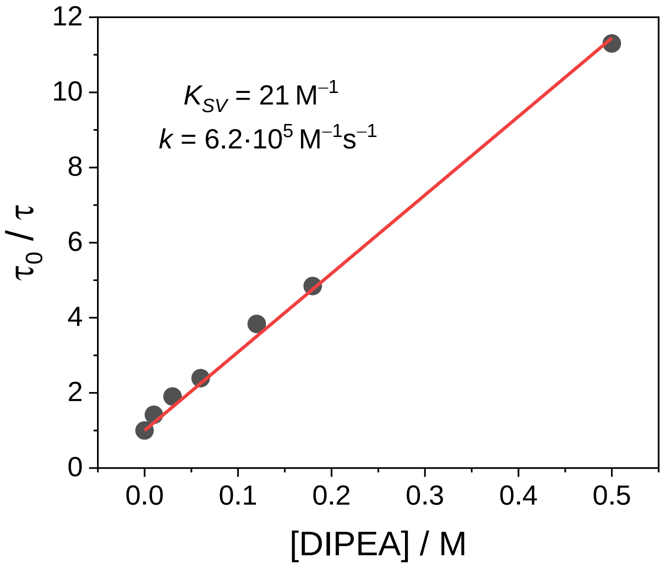

Figure S19. Stern-Volmer analysis of the quenching of the delayed fluorescence of 4-DPAIPN by DIPEA measured by laser-flash photolysis (excitation at 355 nm).

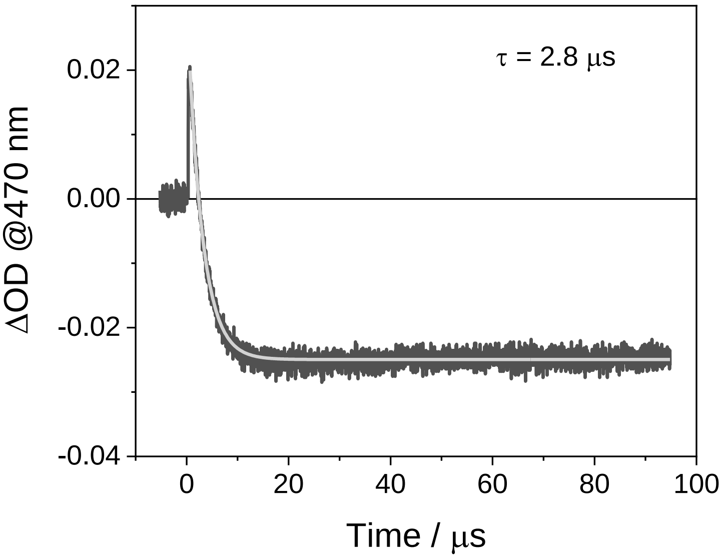

a)

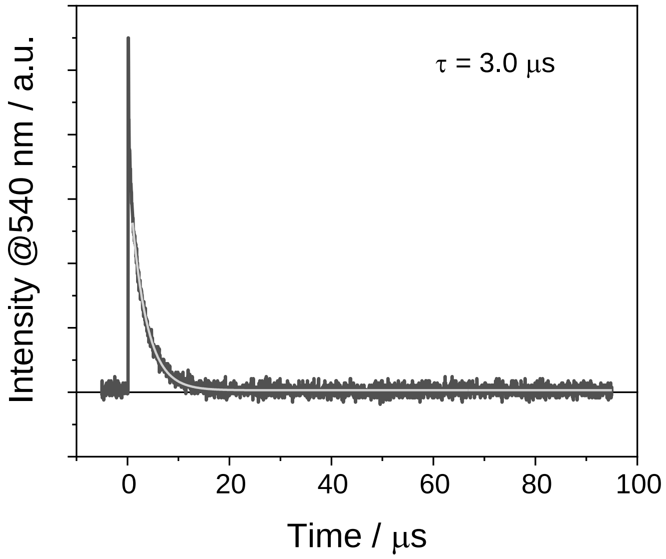

b)

Figure S20. a) Transient absorption decay at 470 nm and b) time-resolved luminescence decay at 540 nm obtained by laser-flash photolysis (excitation at 355 nm) of 25 μM 4-DPAIPN in N_2-purged acetonitrile in the presence of 0.5 M DIPEA.

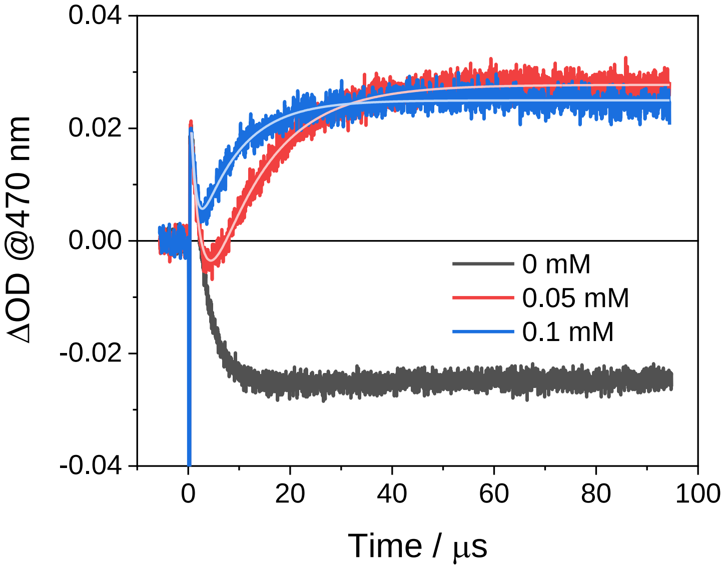

Figure S21. Kinetic traces at 470 nm obtained by laser-flash photolysis (excitation at 355 nm) of 25 μM 4-DPAIPN in N_2-purged acetonitrile in the presence of 0.5 M DIPEA and 0-0.1 mM **FeL**$^{\mathbf{MeOH}}$.

Determination of the bimolecular rate constant

Laser flash photolysis has been employed to follow the reaction involving the photogenerated 4-DPAIPN radical anion and the **FeL^{MeOH}** catalyst (eq. S8).

4-DPAIPN + hν → 4-DPAIPN* (S6)

4-DPAIPN* + DIPEA → 4-DPAIPN^{-} + DIPEA^{+} (S7)

4-DPAIPN^{-} + Fe(II) → 4-DPAIPN + Fe(I) (S8)

Formation of Fe(I) is monitored by transient absorption spectroscopy from the development of an absorption above 600 nm following the reaction sequence in eqs. S6-S8. The kinetic traces in Figure 3 of the main text have been fitted using a biexponential function. The first (decaying) component is assigned to eq. S7, while the second (rising) component to eq. S8. This latter has been thus employed to estimate the bimolecular rate constant for the reaction in eq. S8.

Under pseudo-first order kinetic conditions (i.e., [Fe(II)] >> [4-DPAIPN^{-}]), the rate of Fe(I) formation can be described according to eqs. S9-S11.

$$\frac{d[\boldsymbol{Fe(I)}]}{dt} = -\frac{d[\boldsymbol{4DPAIPN^{-}}]}{dt} = k\,[\boldsymbol{4DPAIPN^{-}}][\boldsymbol{Fe(II)}] \approx k'[\boldsymbol{4DPAIPN^{-}}] \quad \text{(S9)}$$

$$k' = k[\boldsymbol{Fe(II)}] \quad \text{(S10)}$$

$$[\boldsymbol{4DPAIPN^{-}}]_t = [\boldsymbol{4DPAIPN^{-}}]_0\, exp(-k't) \quad \text{(S11)}$$

The evolution of the transient absorption at 750 nm can be thus described according to eq. S12 that takes into account the mass balance of the reaction (eq. S13).

$$[\boldsymbol{Fe(I)}]_t = [\boldsymbol{4DPAIPN^-}]_0 - [\boldsymbol{4DPAIPN^-}]_0\, exp(-k't) =$$

$$= [\boldsymbol{4DPAIPN^-}]_0\, \{1 - exp(-k't)\} \quad \text{(S12)}$$

$$[\boldsymbol{4DPAIPN^-}]_0 = [\boldsymbol{4DPAIPN^-}]_t + [\boldsymbol{Fe(I)}]_t \quad \text{(S13)}$$

From the Lambert-Beer law, the fitting according to the eq. S14 allows to extract the pseudo-first order rate constant k'. Knowing the Fe(II) concentration and using eq. S10, the bimolecular rate constant k can be thus determined (Figure S21).

$$\Delta OD(750)_t = \Delta OD(750)_0\, \{1 - exp(-k't)\} \quad \text{(S14)}$$

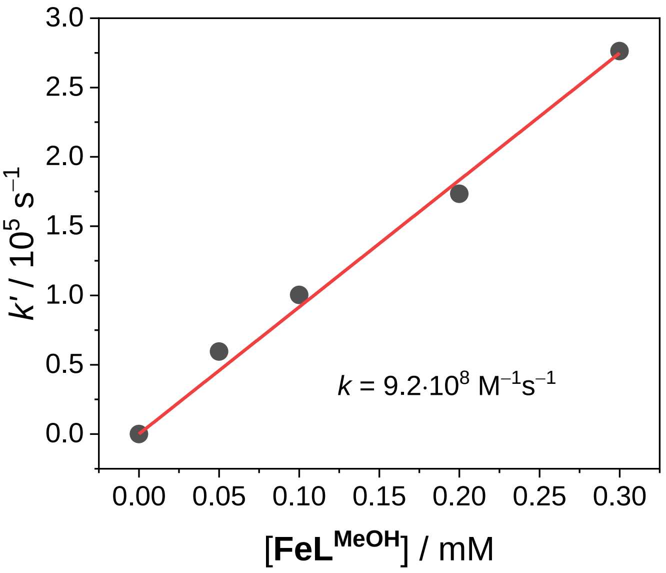

Figure S22. Plot of the observed rate constant k' of Fe(I) formation vs the concentration of $\mathbf{FeL^{MeOH}}$ for the determination of the bimolecular rate constant k.

5. References of the SI

[S1] A. Juris, V. Balzani, F. Barigelletti, S. Campagna, P. Belser, A. Von Zelewsky, *Coord. Chem. Rev*. **1988**, *84*, 85-277.

[S2] A. Vega-Peñaloza, J. Mateos, X. Companyó, M. Escudero-Casao, L. Dell'Amico, *Angew. Chem. Int. Ed.* **2021**, *133*, 1096-1111.

[S3] S. P. Pitre, C. D. McTiernan, W. Vine, R. DiPucchio, M. Grenier, J. C. Scaiano, *Sci. Rep.* **2015**, *5*, 16397.

[S4] F. Droghetti, F. Lemken, L. Rulisek, A. Ruggi, M. Natali, *ACS Catal*. **2024**, 14, 16920-16935.

[S5] P. De La Torre, J. S. Derrick, A. Snider, P. T. Smith, M. Loipersberger, M. Head-Gordon, C. J. Chang, *ACS Catal*. **2022**, *12*, 8484-8493.

[S6] P. Y. Ho, S. C. Cheng, F. Yu, Y. Y. Yeung, W. X. Ni, C. C. Ko, C. F. Leung, T. C. Lau, M. Robert, *ACS Catal.* **2023**, *13*, 5979-5985.

[S7] J. W. Wang, Z. Li, Z. M. Luo, Y. Huang, F. Ma, S. Kupfer, G. Ouyang, *Proc. Natl. Acad. Sci. U.S.A.* **2023**, *120*, e2221219120.

[S8] F. Ma, Z. M. Luo, J. W. Wang, G. Ouyang, *J. Am. Chem. Soc*. **2024**, *146*, 17773-17783.

[S9] Z. Guo, G. Chen, C. Cometto, B. Ma, H. Zhao, T. Groizard, L. Chen, H. Fan, W. L. Man, S. M. Yiu, K. C. Lau, T. C. Lau, M. Robert, *Nat. Catal*. **2019**, *2*, 801-808.
